# The Functional *OAS1* rs10774671A>G Variant Is Associated with COVID-19 Susceptibility in Mexican Patients

**DOI:** 10.3390/ijms27072965

**Published:** 2026-03-25

**Authors:** Isela Montúfar-Robles, Blanca M. Zapotitla-Román, Gilberto Vargas-Alarcón, José Manuel Fragoso, Silvia Jiménez-Morales, Jorge Flavio Mendoza-Rincón, Alberto Cedro-Tanda, Rosa Elda Barbosa-Cobos, Gustavo Rojas-Velazco, Julian Ramírez-Bello

**Affiliations:** 1 División de Investigación, Hospital Juárez de México, Mexico City 07760, Mexico; ismontufar@gmail.com; 2 Subdirección de Investigación Clínica, Instituto Nacional de Cardiología Ignacio Chávez, Mexico City 14080, Mexico; zapotitlablanca4@gmail.com; 3 Dirección de Investigación, Instituto Nacional de Cardiología Ignacio Chávez, Mexico City 14080, Mexico; gvargas63@yahoo.com; 4 Laboratorio de Biología Molecular, Instituto Nacional de Cardiología Ignacio Chávez, Mexico City 14080, Mexico; mfragoso1275@yahoo.com.mx; 5 Laboratorio de Innovación y Medicina de Precisión Núcleo A, Instituto Nacional de Medicina Genómica, Mexico City 14610, Mexico; sjimenez@inmegen.gob.mx; 6 Laboratorio de Oncología Molecular, L9 (UMIEZ), Campus II, FES-Zaragoza-UNAM, Mexico City 09230, Mexico; jflavio.m@gmail.com; 7 Núcleo B de Innovación en Medicina de Precisión, Instituto Nacional de Medicina Genómica, Mexico City 14610, Mexico; acedro@inmegen.gob.mx; 8 Servicio de Reumatología, Hospital Juárez de México, Mexico City 07760, Mexico; rebcob@yahoo.com; 9 Unidad de Cuidados Intensivos, Instituto Nacional de Cardiología Ignacio Chávez, Mexico City 14080, Mexico; gustavortic@gmail.com

**Keywords:** SARS-CoV-2, COVID-19, susceptibility, severity, SNV

## Abstract

*OAS1* (2′–5′-oligoadenylate synthetase 1) and *OAS3* have been identified through a genome-wide association study as major *loci* associated with COVID-19. The rs10774671A>G variant affects alternative splicing and generates two distinct mRNA and protein isoforms. The A allele produces the shorter p42 isoform, which has been associated with increased susceptibility, greater disease severity, and higher mortality from COVID-19, whereas the G allele produces the longer p46 isoform, which has been associated with a protective effect. In addition, the functional variants *OAS1* rs4767027C>T, OAS1 rs1131454A>G, and *OAS3* rs10735079A>G have also been associated with susceptibility to and/or severity of COVID-19. Therefore, the aim of this study was to determine whether four variants in the *OAS1* and *OAS3* genes are associated with susceptibility to COVID-19 and with the clinical signs and symptoms of the disease. We included 305 patients with COVID-19 and 288 healthy controls. We genotyped the *OAS1* rs10774671A>G, rs4767027C>T, rs1131454A>G, and *OAS3* rs10735079A>G variants using TaqMan^®^ assays. The association between *OAS1* and *OAS3* variants and disease susceptibility or severity was assessed using binary logistic regression adjusted for age and sex. The Hardy–Weinberg equilibrium was evaluated using SNPStats, whereas haplotypes and linkage disequilibrium were analyzed with Haploview. Statistical power was calculated using Quanto. Logistic regression analysis adjusted for age and sex revealed an association between the *OAS1* rs10774671A risk allele and susceptibility to COVID-19 (G vs. A: OR = 1.9, *p* = 0.007). In contrast, no associations with COVID-19 susceptibility were observed for the rs4767027C>T, rs1131454A>G, or rs10735079A>G variants. However, the rs1131454A>G and rs10735079A>G variants showed associations with sore throat. Overall, our findings suggest that *OAS1* acts as a susceptibility factor for COVID-19 and the rs1131454A>G and rs10735079A>G SNVs are associated with sore throat in the Mexican population.

## 1. Introduction

In December 2019, several patients in Wuhan, China, presented with atypical pneumonia, and the causative agent was later identified as severe acute respiratory syndrome coronavirus 2 (SARS-CoV-2), responsible for coronavirus disease 2019 (COVID-19) [1,2,3]. SARS-CoV-2 infects human epithelial and endothelial cells through its spike (S) protein, which binds to the angiotensin-converting enzyme 2 receptor [4,5,6]. Host proteases, including TMPRSS2, ELANE, and cathepsin L, cleave the S protein, facilitating viral entry into host cells [7]. After entry, the positive-sense single-stranded RNA (ssRNA) genome is released into the cytoplasm, where it recruits host ribosomes for the translation of open reading frame (ORF) 1a and ORF1b. Portions of the viral genome are subsequently replicated into double-stranded RNA (dsRNA), which is produced early during infection as a result of genome replication and subgenomic mRNA transcription [8,9]. Viral dsRNA activates the 2′–5′-oligoadenylate synthetase (OAS)–ribonuclease L (RNase L) pathway, an essential component of the innate immune response against SARS-CoV-2 [10]. Activation of OAS leads to RNase L-mediated degradation of viral RNA, thereby limiting viral replication [11].

In 2021, a single-nucleotide variant (SNV) located within the *OAS1–OAS3* gene cluster was identified as a risk factor for COVID-19 through a genome-wide association study (GWAS) conducted in European populations [12]. Subsequently, other studies reported associations between several *OAS1–OAS3* SNVs and COVID-19 susceptibility or severity in individuals of European, admixed American, African, Middle Eastern, South Asian, and East Asian ancestry [13,14,15]. One of the most extensively studied variants is *OAS1* rs10774671A>G, which affects the splice acceptor site located at the last nucleotide of intron 5. This variant results in the generation of two distinct *OAS1* protein isoforms: the rs10774671A allele produces a shorter p42 isoform, whereas the G allele generates a longer p46 isoform with higher enzymatic activity [16,17]. The rs10774671G allele has been associated with resistance to West Nile virus infection [18], a more favorable response to interferon (IFN) treatment in individuals infected with hepatitis C virus [19], and protection against susceptibility, severity, and hospitalization in patients with COVID-19 [14,20,21]. In contrast, the rs10774671A allele has shown no detectable anti-SARS-CoV-2 activity and has been associated with increased susceptibility to COVID-19, greater disease severity, and higher risk of hospitalization [22,23,24].

In addition, other SNVs, including *OAS1* rs4767027C>T, rs1131454A>G (Gly162Ser), and rs10735079A>G, have also been associated with COVID-19, primarily in individuals of European ancestry; however, populations with different ancestral backgrounds have been far less evaluated. In Mexico, two independent studies have reported a high frequency of the rs10774671A allele in populations from Monterrey, Nuevo León (northeastern Mexico), and Zacatecas (northern Mexico) [25,26]. A study conducted in patients from Zacatecas reported no association between this variant and hospitalization status (hospitalized vs. non-hospitalized patients), clinical outcome (recovered vs. deceased patients), or disease severity [26]. In Mexico and in most Latin American populations, this variant has been largely unexplored in patients with COVID-19. Specifically, the variants rs10774671A>G, rs4767027C>T, rs1131454A>G, and rs10735079A>G have not been evaluated for their association with susceptibility to COVID-19 or with specific clinical signs and symptoms of the disease. The selection of the *OAS1* and *OAS3* variants was based on the following criteria: (a) variants previously associated with COVID-19 in several populations, particularly in Europe and Africa; (b) variants with relatively high allele frequencies in different populations; and (c) variants with reported functional relevance. Specifically, rs10774671A>G affects the splicing of OAS1; rs4767027C>T has been suggested to act as a protein quantitative trait locus (pQTL) for OAS1; rs1131454A>G (Gly162Ser) is a missense variant reported to regulate OAS1 expression (including both the p46 and p42 isoforms) through a splicing enhancer mechanism; and the *OAS3* rs10735079A>G variant has been associated with reduced circulating levels of both OAS3 and OAS1. Therefore, in the present study, we evaluated whether three *OAS1* SNVs (rs10774671A>G, rs1131454A>G [Gly162Ser], and rs4767027C>T) and one *OAS3* SNV (rs10735079A>G) are associated with susceptibility to COVID-19 and with selected clinical manifestations of the disease in individuals from Central Mexico.

## 2. Results

### 2.1. Clinical Signs and Symptoms in Patients with COVID-19

Table 1 summarizes the clinical signs, symptoms, and comorbidities observed in patients with COVID-19. Cough, dyspnea, need for mechanical ventilation, myalgia, and fatigue were the most prevalent clinical manifestations among the patients.

### 2.2. Hardy–Weinberg Equilibrium (HW-e) and Statistical Power

After analyzing the genotype frequencies of *OAS1* rs10774671A>G, rs1131454A>G (Gly162Ser), rs4767027C>T, and *OAS3* rs10735079A>G in the control group, no deviation from HW-e was observed (*p* > 0.05). Our statistical analysis indicated that, based on the parameters outlined in the power calculation, the statistical power for each evaluated *OAS1* and *OAS3* variant was less than 80%.

### 2.3. Allele and Genotype Frequencies of OAS1 and OAS3 SNVs in Cases and Controls and Association Analysis

Our data show that the *OAS1* rs10774671A>G variant, analyzed by binary logistic regression and adjusted for age and sex, is a risk factor for COVID-19 under the allelic (G vs. A; OR = 1.91, *p* = 0.007) and recessive (GG + GA vs. AA; OR 2.14, *p* = 0.007) models (Table 2). The functional rs10774671A allele showed a frequency of 77.7% in controls and 85.4% in cases and was the only variant associated with susceptibility to COVID-19 in our study population (Table 2). In contrast, the remaining *OAS1* variants and the *OAS3* variant did not show an association with COVID-19, as no statistically significant differences were observed in either allele or genotype frequencies between cases and controls. Therefore, these three variants were not identified as risk factors for developing COVID-19 in our study population. (Table 2). Allele and genotype frequencies in cases and controls for these three variants, along with odds ratios (ORs), 95% confidence intervals (CIs), and *p*-values, are presented in Table 2.

On the other hand, the frequency of the rs10774671A allele in Mexican individuals free of SARS-CoV-2 infection and without COVID-19, as reported by the 1000 Genomes Project [27], by Sánchez-González et al. [25], and by our research group, is shown in Table 3. Comparison of allele frequencies across these three populations revealed no statistically significant differences (Table 3). In addition, we also compared the frequency of the *OAS1* rs10774671A allele between our COVID-19 patients and the individuals included in the Sánchez-González study, as well as with the published data from the 1000 Genomes Project. This analysis revealed a trend toward association between our cases and the individuals from Nuevo León (OR 1.50, 95% CI 0.99–2.28, *p* = 0.052), whereas a statistically significant difference was observed between our cases and the Mexican individuals living in Los Angeles (OR 2.12, 95% CI 1.35–3.33, *p* = 0.001) (Table 3).

### 2.4. Haplotypes and LD Among OAS1 and OAS3 SNVs in Cases and Controls

We identified four haplotypes composed of *OAS1* and *OAS3* variants with frequencies greater than 1% in both cases and controls: AACA, GGTG, GACA, and GGCA. The GGTG haplotype, which carries the minor alleles of rs1131454A>G, rs10774671A>G, rs4767027C>T, and rs10735079A>G, was significantly associated with protection against COVID-19 (OR = 0.54; permutation-corrected *p*-value [*pc*] = 0.005 based on 10,000 permutations. In contrast, the AACA haplotype, formed by the common alleles of these four variants, showed no association with susceptibility to COVID-19 in our study population (*p* > 0.05).

**Figure 1:**
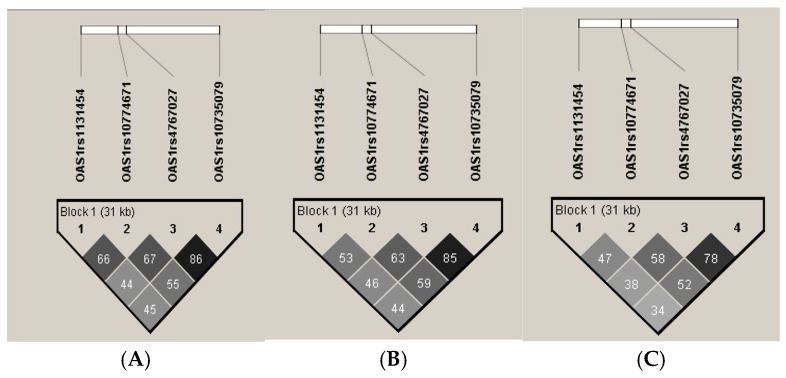
Linkage disequilibrium (LD) among *OAS1* and *OAS3* variants in patients with COVID-19, Mexican individuals living in Los Angeles, and our control group. (**A**) LD among the four *OAS1* and *OAS3* variants in Mexican patients infected with SARS-CoV-2 who developed COVID-19 and in our control group, (**B**) LD among individuals of Mexican ancestry living in Los Angeles without SARS-CoV-2 infection, (**C**) and LD among our control group. (**A**,**B**) show that the rs4767027C>T and rs10735079A>G variants exhibited the highest LD. Darker colors indicate stronger LD values, whereas lighter colors indicate weaker LD. Typically, an r^2^ value close to 1 (often displayed as black) indicates a strong correlation between SNPs, meaning that the alleles are frequently inherited together. Intermediate colors represent moderate LD, while light colors or white squares correspond to low r^2^ values, indicating little or no correlation between variants.

Regarding the LD analysis, none of the four *OAS1* and *OAS3* variants showed strong LD in either cases or controls. The highest r^2^ value was observed between the rs4767027C>T and rs10735079A>G variants (Figure 1A–C). This finding is consistent with data reported in the Ensembl LD database for Mexican individuals from Los Angeles (MXL population), where these two variants also show the highest degree of LD (Figure 1B) [28].

### 2.5. Analysis of OAS1 and OAS3 Variants and Clinical Traits

Analyses of *OAS1* and *OAS3* variants in relation to the clinical signs and symptoms of COVID-19 (Table 1), evaluated using binary logistic regression and adjusted for age and sex, showed that the rs10774671A>G and rs4767027C>T variants were not associated with any clinical features of the disease (*p* > 0.05) In contrast, the rs1131454A>G and rs10735079A>G variants showed significant associations with odynophagia. Specifically, under the codominant model, the OAS1 rs1131454A>G variant (GG vs. GA: OR = 0.22, *p* = 0.017) and the OAS3 rs10735079A>G variant (GG vs. GA: OR = 0.11, *p* = 0.012; GG vs. AA: OR = 0.19, *p* = 0.04) exhibited a protective effect.

## 3. Discussion

*OAS1*, which encodes the 2′–5′-oligoadenylate synthetase 1 enzyme, has been identified as an important gene involved in COVID-19 susceptibility and severity [12,13,14,15]. OAS1 is an interferon-inducible antiviral protein that mediates activation of RNase L, resulting in the degradation of SARS-CoV-2 RNA and inhibition of viral replication [29,30]. A functional *OAS1* variant located in intron 5 (rs10774671A>G) affects the splice acceptor site, leading to the production of two distinct *OAS1* isoforms. Specifically, the rs10774671A allele generates a shorter p42 isoform, whereas the G allele produces a longer p46 isoform with higher enzymatic activity [16,17]. The rs10774671G allele has been associated with protection against West Nile virus infection [18], with improved response to interferon therapy in individuals infected with hepatitis C virus [19], and with reduced susceptibility, severity, and hospitalization in patients with COVID-19 [14,20,21]. In contrast, the rs10774671A allele has been reported to show little or no antiviral activity against SARS-CoV-2 and has been associated with increased susceptibility to COVID-19, greater disease severity, and higher hospitalization rates [22,23,24,31].

Regarding the frequency of the *OAS1* rs10774671A allele, it has been reported to occur at lower frequencies in African populations (29.8–38.9%) compared with American (62.0–88.8%), European (58.4–71.7%), and Asian (64.6–83.7%) populations [27], based on data from individuals included in the 1000 Genomes Project. Similar allele and genotype frequencies for this variant have been described among individuals with European, African, and Amerindian ancestry [14,15,25]. In Mexican individuals without SARS-CoV-2 infection and living in Los Angeles, the frequency of the rs10774671A allele is reported to be 73.4% [27]; meanwhile, in people from Nuevo León (located in northeast Mexico), it was 79.6% [25], and in our current study, that includes healthy individuals from Central Mexico, it was 77.7%. Another Mexican research group that included only symptomatic and asymptomatic COVID-19 patients from Zacatecas (northern Mexico) reported an A allele frequency of 96.2% [26]. Overall, the rs10774671A allele frequency observed in our study was comparable to that reported in Ensembl for Mexicans living in Los Angeles, California [27], as well as to frequencies described by Sánchez-González et al. [25]. Collectively, the high frequency of the *OAS1* rs10774671A allele in individuals from Zacatecas, Nuevo León, and Central Mexico suggests that a substantial proportion of the population carries this risk allele, which contributes to increased susceptibility to COVID-19. Our results suggest that the rs10774671A>G variant is a risk factor for COVID-19 susceptibility in the Mexican population. This finding is consistent with previous studies conducted in Ecuadorian, European, and African populations, in which the A allele or the GA/AA genotypes have been associated with increased susceptibility to COVID-19 and/or greater disease severity [14,15,31]. Therefore, our results provide additional evidence supporting the role of the *OAS1* rs10774671A>G variant in COVID-19 susceptibility in a population with significant Amerindian ancestry.

In addition, we evaluated three additional variants in *OAS1* (rs4767027C>T and rs1131454A>G (Gly162Ser) and *OAS3* (rs10735079A>G), which have been reported to be associated with COVID-19 [12,15]. After analysis, we did not identify any association between these *OAS1* and *OAS3* variants and susceptibility to COVID-19. All three variants have been reported to play potentially relevant biological roles in *OAS1* and *OAS3*. For example, the *OAS1* rs4767027C>T variant has been suggested to act as a protein quantitative trait locus (pQTL) and may influence circulating protein levels [14]. Specifically, the C allele (which is in linkage with the rs10774671-A risk allele) has been associated with lower levels of the p46 isoform of OAS1, thereby contributing to increased susceptibility to this infectious disease [14]. Meanwhile, rs1131454A>G, a missense variant located in exon 3 that results in a glycine-to-serine substitution at position 162, has been reported to regulate *OAS1* expression (both the p46 and p42 isoforms) through a splicing enhancer mechanism. In this context, the rs1131454G allele creates a putative exonic splicing enhancer/silencer (ESE/ESS), whereas the rs1131454A allele disrupts this regulatory element [15]. In addition, the rs10735079A>G variant—one of the first variants in the *OAS1–OAS3* genomic region reported to be associated with susceptibility to COVID-19 in Caucasian populations and which is in high linkage disequilibrium (r^2^ = 0.87) with rs10774671A>G—is located in intron 2 of *OAS3* and has also been reported to have functional relevance. Specifically, the rs10735079A risk allele has been associated with reduced circulating levels of OAS3 [32]. Two recent studies have further shown that rs10735079A>G affects *OAS1* gene expression levels in monocytes [33], while another study reported an association with decreased *OAS1* expression [34].

Notably, these three *OAS1* and *OAS3* variants have been scarcely studied worldwide, with reported associations mainly in European and African populations [12,14,15]. However, to the best of our knowledge, these variants have not been evaluated in populations enriched for Amerindian ancestry. One of the first GWASs in patients infected with SARS-CoV-2 who developed COVID-19 reported rs10735079A>G as a risk factor for this viral disease [12]. Nevertheless, this variant has been scarcely evaluated in other COVID-19 patient cohorts; studies conducted in individuals from Morocco and Palestine found no association with disease severity or hospitalization [35,36]. Our results are consistent with these last studies of no association between this *OAS3* variant and susceptibility to COVID-19. Regarding the rs1131454A>G variant, which has been associated with COVID-19 in European, African, and Japanese populations [15,37,38], to the best of our knowledge, no additional studies have evaluated this variant in the context of this viral infectious disease. Our data indicate no association between rs1131454A>G and susceptibility to COVID-19 in the Central Mexican population. On the other hand, the rs4767027C>T variant has been associated with COVID-19 severity in patients from European and African populations [14,15,39]. However, we did not identify any association between this variant and susceptibility to COVID-19. The discrepancies between previously reported associations of the *OAS1* variants rs1131454A>G and rs4767027C>T with COVID-19 susceptibility and the lack of association observed in our study population may be attributable to differences in sample size, population structure, ethnic background, or other unmeasured factors.

On the other hand, we did not identify any association between the *OAS1* rs10774671A>G variant and the range of clinical symptoms and signs presented by COVID-19 patients in our study. Previously, a study conducted in a Mexican population from Zacatecas reported no association between this variant and multiple clinical manifestations, including fever, cough, headache, dyspnea, chest pain, myalgia, rhinorrhea, odynophagia, among others, in patients with COVID-19 [26]. Our findings are consistent with those previously reported in patients from Zacatecas. We also found no association between the rs4767027C>T variant and the clinical symptoms or signs of COVID-19. It is important to note that a limitation of both studies is the sample size; therefore, analyses in larger cohorts are required to obtain more robust and reliable results.

In our study, the *OAS1* rs1131454A>G and *OAS3* rs10735079A>G variants were associated with protection against the development of odynophagia in patients with COVID-19. A recent study reported that carriers of the *OAS3* rs10735079A allele were less likely to develop muscle pain and sore throat. In our analysis, we identified a protective OR for sore throat during swallowing (odynophagia). Our findings are consistent with those reported in the Palestinian population. In contrast, we did not replicate the previously reported association between the rs10735079A allele and muscle pain [36]. On the other hand, we found that the rs1131454A>G variant was also associated with odynophagia. To the best of our knowledge, no previous studies have evaluated this specific symptom in relation to this variant in patients with COVID-19. Identifying genetic variants associated with specific clinical signs or symptoms of COVID-19 could provide valuable information for understanding individual variability in disease presentation. In this context, identifying individuals who carry certain genotypes of the *OAS1* rs1131454A>G and *OAS3* rs10735079A>G variants may help predict the likelihood of developing particular clinical manifestations of COVID-19, such as odynophagia. However, given the limited available evidence, no definitive conclusions can be drawn regarding the relationship between rs1131454A>G and odynophagia. Further studies involving larger patient cohorts are required to clarify the role of this variant in this clinical manifestation.

Our study has several limitations that should be considered when interpreting the results. First, although a relatively balanced number of cases and controls was included, the overall sample size remains moderate. Consequently, the statistical power of our study was below the commonly recommended threshold of 80%, which is generally recommended to reliably detect variants with modest effect sizes. Studies with lower statistical power increase the risk of type II errors, meaning that true associations may fail to reach statistical significance [40]. Therefore, our ability to detect variants with small effect sizes may be limited, and the findings should be interpreted with caution. Second, another limitation of our study is that ancestry-informative markers (AIMs) were not evaluated. Therefore, it was not possible to assess the genetic ancestry or population structure of the study population. Population stratification may lead to spurious associations or may mask true genetic effects if cases and controls differ in their ancestral background [41]. This issue is particularly relevant in admixed populations, such as the Mexican population, where allele frequencies may vary substantially among ancestral groups. Without accounting for population structure, the observed genetic associations could potentially reflect underlying ancestry differences rather than true disease susceptibility *loci*. Consequently, the potential influence of population stratification cannot be completely ruled out when interpreting our results. Third, our analysis was restricted to three variants in the *OAS1* gene and one variant in the *OAS3* gene, which does not allow a comprehensive assessment of other potentially functional or regulatory variants within these genes or in related antiviral pathways. Fourth, another limitation that could introduce bias in our study is the selection of the control group. We acknowledge that using healthcare workers as controls may introduce certain limitations, since they may not be fully representative of the general population. However, this study design was intentionally chosen to include individuals with a high probability of exposure to SARS-CoV-2 who nevertheless did not develop infection. This approach may help reduce misclassification of exposure status and allows the identification of genetic factors potentially associated with resistance to infection. Nevertheless, we recognize that differences in occupational exposure, protective behaviors, and other environmental factors may exist between healthcare workers and the general population. Therefore, the possibility of selection bias cannot be completely excluded. Finally, our findings should be replicated in independent cohorts with larger sample sizes. In particular, the association observed between the *OAS1* rs10774671A>G variant and susceptibility to COVID-19, as well as the associations between the *OAS1* rs1131454A>G and *OAS3* rs10735079A>G variants and protection against the development of odynophagia in patients with COVID-19, should be evaluated in additional populations. Replication studies in individuals from Central Mexico and other regions of the country would help determine whether these *OAS1* and *OAS3* variants represent genetic factors associated with susceptibility to or severity of COVID-19. Furthermore, replication in other Latin American populations would be particularly relevant, given the high degree of genetic admixture in these populations, including a substantial Amerindian ancestry component.

## 4. Materials and Methods

### 4.1. Study Population Characteristics

A total of 305 patients infected with SARS-CoV-2 and 288 control individuals from Central Mexico were recruited. SARS-CoV-2 infection status was confirmed by reverse transcription polymerase chain reaction. Sample collection for both patients with COVID-19 and controls was conducted between April 2020 and February 2021 at Hospital Juárez de México (HJM) and the Instituto Nacional de Cardiología Ignacio Chávez (INCICh). The diagnosis of COVID-19 was based on the presence of clinical signs and symptoms, including loss of taste and smell, dry cough, dyspnea, myalgia, fatigue, fever, headache, odynophagia, diarrhea, vomiting, abdominal pain, body temperature, oxygen saturation, and heart rate. In addition, a positive RT-PCR test for SARS-CoV-2 and/or evidence of atypical pneumonia on chest computed tomography scans was used to further confirm the diagnosis. The exclusion criteria for patients were as follows: individuals younger than 24 years of age, non-Mexican ancestry, receipt of a blood transfusion within the previous two weeks, pregnancy, and death prior to blood sample collection. Cases included individuals hospitalized with severe or critical COVID-19 (requiring admission to the intensive care unit [ICU], high-flow oxygen therapy, or mechanical ventilation), as well as individuals with asymptomatic or mild disease. The control group consisted of 288 healthy individuals older than 24 years, recruited from the INCICh. All controls were healthcare workers actively involved in the care of patients with COVID-19 in the ICU, including medical residents, laboratory personnel, and nurses. To confirm the absence of prior SARS-CoV-2 infection, all controls underwent serological testing for SARS-CoV-2 antibodies using the Elecsys^®^ Anti-SARS-CoV-2 assay (Roche Diagnostics International Ltd., Rotkreuz, Switzerland).

### 4.2. Nuclear DNA Isolation and Genotyping of Genetic Markers

Buffy coat fractions were obtained from peripheral blood collected in purple-top tubes containing K2-EDTA (BD Vacutainer, Franklin Lakes, NJ, USA) by centrifugation at 2500 rpm for 10 min at room temperature. Nuclear DNA was subsequently isolated for genetic variant analyses. All procedures were performed within a class II biological safety cabinet (Thermo Fisher Scientific, USA), in accordance with institutional biosafety protocols and the applicable Official Mexican Standards. Briefly, the buffy coat was mixed with 1.5 mL of red blood cell (RBC) lysis buffer (Qiagen, Hilden, Germany), followed by centrifugation and homogenization. Subsequently, 1 mL of white blood cell (WBC) lysis buffer (Qiagen, Germany) was added, and samples were incubated at 60 °C. Genomic DNA was precipitated using chilled absolute (100%) ethanol (Merck, Darmstadt, Germany), washed with 70% ethanol (Merck, Germany) to remove contaminants, and air-dried in a clean tube at 60 °C for 20 min [42]. DNA concentration was quantified with a NanoDrop One/OneC spectrophotometer (Thermo Scientific, Waltham, MA, USA) and samples were diluted to a final concentration of 10 ng/μL for downstream genotyping analyses.

Genotyping of the rs10774671A>G, rs10735079A>G, rs1131454A>G (Gly162Ser), and rs4767027C>T variants was performed using TaqMan^®^ SNP Genotyping Assays on a 7900HT Fast Real-Time PCR System (Applied Biosystems, Foster City, CA, USA). To ensure the quality and reproducibility of the genotyping data, approximately 70% of the samples from both the case and control groups were randomly selected and subjected to independent repeat genotyping as part of the quality control procedures. The repeat genotyping was performed using the same method and under identical experimental conditions as the initial analysis. The concordance rate between the original and repeated genotyping results was 100%, demonstrating a high level of accuracy and reliability of the genotyping data.

### 4.3. Ethical Statement

This study was conducted in accordance with the principles of the Declaration of Helsinki and was approved by the Research, Ethics, and Biosecurity Committees of the INCICh and the HJM under project numbers 21-1237 and HJM 024/22-I, respectively. Written informed consent was obtained from all participants or, when applicable, from their legally authorized representatives, in accordance with institutional guidelines.

### 4.4. Statistical Analysis

Statistical analyses were performed using SPSS software version 18.0 (SPSS Inc., Chicago, IL, USA). The genotype frequencies of each variant were tested for compliance with HW-e in the control group using the SNPStats web-based software (https://www.snpstats.net/start.htm, accessed on 2 January 2026), which evaluates whether the observed genotype frequencies differ significantly from those expected under HW-e by applying a goodness-of-fit test based on allele frequencies. A *p*-value greater than 0.05 was considered indicative of no significant deviation from HW-e. This analysis was performed as a quality control step to assess the reliability of the genotyping data and to verify that the control group was representative of the general population. Allele and genotype frequencies of *OAS1* and *OAS3* SNVs in cases and controls were determined by direct counting. Categorical variables were analyzed using the chi-square test or Fisher’s exact test, when applicable. The association between *OAS1* and *OAS3* SNVs and susceptibility to COVID-19, as well as clinical signs and symptoms, was evaluated using binary logistic regression under different genetic models, with adjustment for age and sex. Results are reported as odds ratios (ORs) with 95% confidence intervals (CIs). A *p*-value ≤ 0.05 was considered statistically significant. Data are presented as mean ± standard deviation (SD). Comparisons among groups were performed using analysis of variance (ANOVA) followed by the least significant difference (LSD) post hoc test, when appropriate. Linkage disequilibrium (LD) and haplotype analyses between OAS1 and OAS3 SNVs were conducted using Haploview software (version 4.2). The statistical power of our study was calculated using the Quanto program (https://keck.usc.edu/biostatistics/software/, accessed on 2 January 2026; Quanto v1.2). The power analysis was performed under the following assumptions: (a) an unmatched case–control study design, (b) a single-gene model, (c) the allele frequencies of the *OAS1* and *OAS3* variants, (d) a dominant genetic model, (e) an odds ratio (OR) of 1.5, and (f) a statistical power of 80%.

## 5. Conclusions

Our results suggest that *OAS1* and *OAS3* may play an important role in susceptibility to and severity of COVID-19 in the Mexican population. Several limitations should be considered when interpreting these findings. In particular, the statistical power of our study was below the commonly recommended threshold of 80%, which may limit our ability to detect variants with small effect sizes. Nevertheless, the genotype distributions in the control group were consistent with HW-e, supporting the reliability of the genetic data analyzed. Therefore, further studies with larger cohorts are needed to confirm these findings. Replication studies in other Mexican populations, as well as in Latin American populations with enriched Amerindian ancestry, would be especially valuable to determine whether variants in *OAS1* and *OAS3* contribute to susceptibility to and/or severity of COVID-19.

## Figures and Tables

**Table 1:** Demographic characteristics and clinical signs and symptoms in patients with COVID-19.

Characteristics	COVID-19 Patients (n * = 305)
Age (years)	55.4 ± 14.2
Sex n (%)	198 (65) Male
	107 (35) Female
Clinical signs and symptoms	n (%)
Cough	195 (64)
Dyspnea	189 (62)
Mechanical ventilation	106 (35)
Myalgia	106 (35)
Fatigue	106 (35)
Fever	82 (27)
Headache	79 (26)
Odynophagia	55 (17)
Diarrhea	39 (12)
Chest Pain	32 (10)
Rhinorrhea	18 (6)
Nausea	15 (5)
Emesis	15 (5)
Abdominal Pain	15 (5)
Temperature °C	36.64 ± 1.04
Oxygen saturation (SpO_2_)	85.03 ± 12.27
Heart rate	89.51 ± 20.12
Comorbidities	n (%)
Obesity	201 (66)
Hypertension	122 (40)
Type 2 diabetes	104 (34)

* n = sample size.

**Table 2:** Genotypic and allelic frequencies of *OAS1* and *OAS3* variants and association analysis in patients with COVID-19 and controls.

SNV	Model	Genotype/ Allele	Controls n (%)	COVID-19 n (%)	OR 95% CI	*p*-Value
	Codominant	GG	17 (6.2)	5 (1.7)		
		GA	89 (32.2)	78 (25.8)	1.52 (0.37–6.15)	0.55
		AA	170 (61.6)	219 (72.5)	3.08 (0.80–11.8)	0.10
rs10774671A>G	Allele	G	123 (22.3)	88 (14.6)		
		A	429 (77.7)	516 (85.4)	1.91 (1.19–3.05)	**0.007**
	Dominant	GG	17 (6.2)	5 (1.7)		
		AG + AA	259 (93.8)	297 (98.3)	2.53 (0.66–9.56)	0.17
	Recessive	GG + AG	106 (38.4)	83 (27.5)		
		AA	170 (61.6)	219 (72.5)	2.14 (1.23–3.73)	**0.007**
rs4767027C>T	Codominant	TT	12 (4.3)	1 (0.3)		
		TC	76 (27.5)	66 (21.9)	4.26 (0.40–44.5)	0.22
		CC	188 (68.1)	235 (77.8)	5.81 (0.57–58.6)	0.13
	Allele	T	100 (18.1)	68 (11.3)		
		C	452 (81.9)	536 (88.7)	1.54 (0.92–2.58)	0.09
	Dominant	TT	12 (4.3)	1 (0.3)		
		TC + CC	264 (95.7)	301 (99.7)	5.36 (0.53–53.8)	0.15
	Recessive	TT + CT	88 (31.9)	67 (22.2)		
		CC	188 (68.1)	235 (77.8)	1.49 (0.85–2.62)	0.16
rs1131454A>G	Codominant	GG	26 (9.4)	17 (5.6)		
		GA	114 (41.3)	119 (39.4)	1.51 (0.54–4.21)	0.42
		AA	136 (49.3)	166 (55.0)	1.71 (0.63–4.63)	0.29
	Allele	G	166 (30.1)	153 (25.3)		
		A	386 (69.9)	451 (74.6)	1.22 (0.82–1.81)	0.32
	Dominant	GG	26 (9.4)	17 (5.6)		
		GA + AA	250 (90.6)	285 (94.4)	1.62 (0.61–4.31)	0.32
	Recessive	GG + AG	140 (50.7)	136 (45.0)		
		AA	136 (49.3)	166 (55.0)	1.20 (0.73–1.98)	0.46
rs10735079A>G	Codominant	GG	10 (3.6)	7 (2.3)		
		AG	82 (29.7)	75 (24.8)	1.42 (0.31–6.37)	0.64
		AA	184 (66.7)	220 (72.8)	1.64 (0.38–7.05)	0.50
	Allele	G	102 (18.5)	89 (14.7)		
		A	450 (81.5)	515 (85.3)	1.20 (0.75–1.91)	0.43
	Dominant	GG	10 (3.6)	7 (2.3)		
		AG + AA	266 (96.4)	295 (97.7)	1.58 (0.37–6.71)	0.53
	Recessive	GG + AG	92 (33.3)	82 (27.2)		
		AA	184 (66.7)	220 (72.8)	1.20 (0.70–2.06)	0.50

SNV: single-nucleotide variant; OR: odds ratio; CI: confidence interval. The *p*-values shown are adjusted for age and sex. *p*-values highlighted in bold indicate statistical significance.

**Table 3:** Frequency of the *OAS1* rs10774671A allele in different Mexican populations.

	Frequency of rs10774671A		*p*-Value
Mexicans (1000 Genomes Project) [27] n (%)	Mexicans from Nuevo León (Northeast Mexico). [25] n (%)	Controls from Central Mexico. Current study n (%)	
94 * (73.4%)	156 ᵆ (79.6%)	429 ^£^ (77.7%)	NS

G vs. A between our cases vs. Mexican individuals who live in Los Angeles: OR 2.12, 95% CI 1.35–3.33, *p* = 0.001. G vs. A between our cases vs. Mexicans from Nuevo León: OR 1.50, 95% CI 0.99–2.28, *p* = 0.052. * 94 out of 128 alleles. ᵆ 156 of 196 alleles ^£^ 429 out of 552. * vs. ᵆ = NS; * vs. ^£^ = NS; ᵆ vs. ^£^ = NS. NS: Not significant.

## Data Availability

The original contributions presented in this study are included in the article. Further inquiries can be directed to the corresponding authors.
